# Glutamate delta-1 receptor regulates oligodendrocyte progenitor cell differentiation and myelination in normal and demyelinating conditions

**DOI:** 10.1371/journal.pone.0294583

**Published:** 2023-11-20

**Authors:** Sukanya G. Gakare, Jay M. Bhatt, Kishore Kumar S. Narasimhan, Shashank M. Dravid

**Affiliations:** Department of Pharmacology and Neuroscience, Creighton University School of Medicine, Omaha, NE, United States of America; Max Planck Institute for Multidisciplinary Sciences, GERMANY

## Abstract

In this study, we investigated the role of glutamate delta 1 receptor (GluD1) in oligodendrocyte progenitor cell (OPC)-mediated myelination during basal (development) and pathophysiological (cuprizone-induced demyelination) conditions. Initially, we sought to determine the expression pattern of GluD1 in OPCs and found a significant colocalization of GluD1 puncta with neuron-glial antigen 2 (NG2, OPC marker) in the motor cortex and dorsal striatum. Importantly, we found that the ablation of GluD1 led to an increase in the number of myelin-associated glycoprotein (MAG+) cells in the corpus callosum and motor cortex at P40 without affecting the number of NG2+ OPCs, suggesting that GluD1 loss selectively facilitates OPC differentiation rather than proliferation. Further, deletion of GluD1 enhanced myelination in the corpus callosum and motor cortex, as indicated by increased myelin basic protein (MBP) staining at P40, suggesting that GluD1 may play an essential role in the developmental regulation of myelination during the critical window period. In contrast, in cuprizone-induced demyelination, we observed reduced MBP staining in the corpus callosum of GluD1 KO mice. Furthermore, cuprizone-fed GluD1 KO mice showed more robust motor deficits. Collectively, our results demonstrate that GluD1 plays a critical role in OPC regulation and myelination in normal and demyelinating conditions.

## Introduction

In the central nervous system (CNS), oligodendrocytes (OLs) are highly specialized glial cells that are responsible for the myelination of axons [1–3]. The OLs are derived from oligodendrocyte precursor cells (OPCs) that have the capacity to proliferate, migrate and differentiate into myelinating OLs [4, 5]. Demyelination is observed in several neurodegenerative diseases, including multiple sclerosis and Alzheimer’s disease as well as in spinal cord and traumatic brain injury and stroke [6–11]. Demyelination results in impairment in nerve conduction leading to severe sensory, cognitive and behavioral symptoms.

Previous studies have shown that OPC differentiation and myelination depend on neural activity [12]. OPCs actively sense neuronal activity in the local environment as they receive synaptic inputs [13–18]. OPCs in both the grey and white matter receive synaptic inputs which can be either glutamatergic or GABAergic [14, 19, 20]. Interestingly, OPCs are the only glial cells that form glutamatergic synapses with axonal projections from neurons (axon-OPC synapses) [4, 19]. Importantly, glutamate signaling in OPCs regulates proliferation, differentiation, and myelination [4, 21–27].

Glutamate delta 1 receptor (GluD1) belong to the delta family of ionotropic glutamate receptors, but they do not function as conventional ligand-gated ion channels [28, 29]. Instead, they form a trans-synaptic complex with presynaptic neurexin (NRXN) through members of cerebellin precursor (Cbln) family [30–32]. Together, the NRXN-Cbln-GluD1 complex regulates synapse formation and maintenance. GluD1 is enriched throughout the forebrain across a variety of neuronal and synaptic subtypes [33–35]. GluD1 controls a variety of behaviors, including sociability, memory, and addiction [35–38]. Using electron microscopy, we have recently characterized ultrastructural expression of GluD1 in the dorsal striatum [39, 40]. In addition to axo-dendritic and axo-spinous locations, GluD1 was also found to be associated with glial processes, but the specific identity of these cells has not been assessed. Interestingly, previous single-cell RNA-seq studies have found that GluD1 expression is highest in OPCs among all CNS cell types [41] (dropviz.org). Moreover, if GluD1 is expressed in OPCs it is possible that owing to its ability to establish NRXN-Cbln-GluD1 trans-synaptic bridge, it may be responsible for the formation and maintenance of axo-OPC synapses [30, 32, 42–44]. GluD1 function in OPCs may also regulate OPC differentiation, OL maturation and subsequent myelination. Interestingly, neurexin has been shown to be critical for the differentiation of OLs and myelin formation in *in vitro* models [45] and neurexin is downregulated in multiple sclerosis and Alzheimer’s disease [46, 47] further supporting a potential role of the trans-synaptic complex.

In this study, we evaluated the role of GluD1 in regulating OPC differentiation and myelination in normal condition and in a cuprizone-induced demyelination model. We found that GluD1 is enriched in neuron-glial antigen 2 positive (NG2+) OPCs. Ablation of GluD1 increased the number of OLs but not NG2+ cells and led to enhanced myelination at P40 (developmental stage). We also found that GluD1 KO mice were more susceptible to behavioral deficits and exhibited susceptibility to demyelination following cuprizone treatment compared to wildtype mice.

## Materials and methods

### Animals

Wildtype (WT) and GluD1 KO male and female mice were used. GluD1 KO mice were obtained from Dr. Jian Zuo [48]. All procedures were approved by the Creighton University Institutional Animal Care and Use Committee and confirmed to the NIH Guide for the Care and Use of Laboratory Animals. Cuprizone (0.2%, bis-cyclohexanone-oxaldihydrazone) diet (TD.140803) and normal diet (2018S) were purchased from ENVIGO (Madison, WI). The mice in the cuprizone group were fed 0.2% w/w cuprizone diet for a total of 5 weeks to induce demyelination. Food pellets were changed twice a week. After 5 weeks of cuprizone treatment, the diet was changed to normal rodent chow for one week to examine remyelination.

### Behavior

#### Rotarod

Motor coordination and balance were evaluated in rotarod test using the Rotamex apparatus (Columbus Instruments, Columbus, Ohio, USA.). The test was carried over three days, two trials per day with an inter-trial interval of 5 hr. On the first day, mice were habituated to the rotarod for 5 min by placing the mice on the rod with no rotation. On second day, the mouse was placed on a rotating rod with a speed of 4 rotations per minute (rpm) for 5 min. On the third day, mice were placed on accelerating rotarod with a starting speed of 4 rpm and reaching 44 rpm in 5 min. The latency (in seconds) to fall or turn completely with the rotating rod was noted by the digital meter of the apparatus. The rotarod test was performed at 0, 5 and 6 weeks.

#### Open field test (OFT)

Open field test was performed in a custom-made square box (38 cm x 38 cm x 30 cm). The mice were placed in the center of the arena and allowed to explore for 60 min. The behavior was videorecorded, and total distance traveled was scored using AnyMaze video-tracking software (Stoelting, Wood Dale, IL, USA). The OFT test was performed at 0, 5 and 6 weeks.

### Immunohistochemistry

Mice were transcardially perfused with 4% PFA in 0.1 M phosphate buffer (PB) and brains were collected and stored overnight in the same fixative at 4°C. Brains were then transferred successively into solutions of 10%, 20% and 30% sucrose in 0.1 M PB before freezing in isopentane at − 30°C to − 40°C. For immunohistochemistry 25 μm thick coronal sections were cut using a cryostat (Leica CM 1900, Buffalo Grove, IL). Sections containing motor cortex, dorsal striatum, hippocampus, carpus callosum were used. After washing 3 times for 5 mins each with 0.1 M PB, sections were incubated in blocking solution containing 10% normal goat and/or donkey serum in 0.25% Triton-X in 0.1 M PB (PBT) for 2 hr at room temperature. Following blocking, sections were incubated overnight at 4°C in the following primary antibodies: guinea pig anti-GluD1 (1:500, GluD1C-GP-Af860, Frontier Institute Co., Ltd, Japan), rabbit anti-NG2 Chondroitin Sulfate Proteoglycan Antibody (1:250, AB5320, Sigma, USA,), rabbit anti-glial fibrillary acidic protein (GFAP, 1:1000, G9269, Sigma, USA) and rabbit anti-ionized calcium-binding adaptor molecule 1 (Iba1, 1:1000, Wako Chemicals 019–19741), mouse anti-myelin associated glycoprotein (MAG, 1:250, sc-166849, SantaCruz Biotechnology, TX, USA), chicken anti-neurofilament protein antibody, heavy chain, 200 KDa (NF-200, 1:1000, NFH, Aves lab, USA), chicken anti-myelin basic protein antibody (1:250, MBP, Aves lab, USA) in PBT. The following day, sections were washed 6 times for 5 mins each in 0.1 M PBT and incubated with the following secondary antibodies: goat anti-guinea pig antibody conjugated to AlexaFluor 488 (1:500, A-11073, Thermo Fisher Scientific), goat anti-rabbit antibody conjugated to AlexaFluor 594 (1:500, A-11012, Life Technologies), goat anti-chicken antibody conjugated to AlexaFluor 594 (1:500, A11042, Life Technologies), donkey anti-mouse antibody conjugated to AlexaFluor 488 (1:500, A21202, Thermo Fisher Scientific, Waltham, MA, USA) for 2 hours at room temperature in dark. Sections were then washed 6 times for 5 mins each in 0.1 M PBT, mounted on pre-cleaned glass slides and coverslipped with Fluoromount-G (0100–01, SouthernBiotech, Birmingham, AL). Images were acquired with Olympus VS120 virtual slide scanning systems or Nikon Ti-E inverted microscope with a Yokagawa spinning disc. For puncta analysis, images of equivalent regions, 1024 × 1024 pixels, were captured using a 60×, oil-immersion objective at a 1× zoom. At least five sections per region of interest were analyzed for each mouse. Morphometric analysis for MBP was carried out by using ImageJ software (NIH). Co-localization of GluD1 with NG2, GFAP and Iba1 were analyzed by Volocity (PerkinElmer Inc. Coventry, United Kingdom). Briefly, puncta (objects) were automatically detected based on the specific fluorophore color, either 594 or 488. Subsequently, thresholding was performed using the standard deviation setting within the Volocity software. These thresholding parameters were individually adjusted and maintained consistently throughout the entire analysis process. Using these established settings, we quantified the total number of puncta for GluD1 and the intensity for MBP, which were expressed as 100%. Additionally, the number of GluD1 puncta colocalized with NG2, GFAP and Iba1 was determined and calculated as the percentage of colocalized puncta to the total puncta. For intensity measurement, we assessed the total intensity in the WT group and considered it as a baseline (100%). The percentage change in intensity in KO mice was then calculated accordingly.

### Synaptoneurosome preparation and western blot analysis

To prepare synaptoneurosomes, mice (P60-80) were first anesthetized with isoflurane and then decapitated. All experimental procedures were conducted on ice. The brain was removed from the skull and rinsed with ice-cold aCSF to remove any adherent blood. The somatosensory cortex (SSC) was then punched and homogenized in a synaptoneurosome buffer (10 mM HEPES, 1 mM EDTA, 2 mM EGTA, 0.5 mM DTT, 0.5 mM PMSF, 50 μg/ml soybean trypsin inhibitor, 0.25% of phosphatase inhibitor cocktail 2% and 3%, and 0.25% of protease inhibitor cocktail) and sonicated with 3 pulses (FB50, Fischer Scientific, Pittsburgh, PA, USA). The resulting sample was filtered twice through three layers of a pre-wetted 100 μm pore nylon filter (CMN-0105-D, Small Parts Inc., Logansport, IN, USA) held in a 13 mm diameter filter holder (XX3001200, Milipore, MA, USA). Then the resulting filtrate was filtered once through a pre-wetted 5 μm pore hydrophilic filter (CMN-0005-D, Small Parts Inc.). The filtrate was centrifuged at 1000 × g for 10 min at 4°C to obtain the synaptoneurosome fraction. Isolated synaptoneurosomes were resuspended in synaptoneurosome buffer containing 0.32 M sucrose, and 1 mM NaHCO3. Protein concentration was measured with Pierce™ BCA Protein Assay Kit (23227, Thermo Fisher Scientific). For western blotting, synaptoneurosomes were loaded on 12% Sodium dodecyl sulfate gel in equal amount (50 μg/well). The samples were run at 100 volts for 90 minutes. Gels were transferred to activated (incubation in methanol) Polyvinylidene Fluoride (PVDF) membrane (GE Healthcare, Piscataway, NJ, USA) at 115 volts for 1 h 45 min. Transfer was followed by blocking with 5% milk in Tris-buffered Saline with 0.1% Tween 20 (TBST) for 1 h at room temperature. The primary antibodies: mouse anti-MAG (#sc-166849, Santacruz Biotechnology), 1:2000; chicken anti-MBP (#MBP, Aves Labs), 1:2000; and mouse anti-β actin (#MA1-140, Invitrogen), 1:3000 was used and kept overnight for incubation at 4°C followed by washing. Blots were incubated with the secondary antibodies for 1 h at room temperature followed by washing with TBST. Blots were incubated with Super- Signal™ West Pico PLUS Chemiluminescent Substrate (34580, ThermoFisher Scientific) and developed in a ChemiDoc imaging system (Bio- Rad Laboratories, Inc., USA). The optical density of each sample was analyzed using ImageJ and normalized to β-actin. Each western blot data point in each group was obtained from a separate animal.

### Statistical analysis

All data are presented as mean ± SEM. Data were analyzed using Student’s parametric two-tailed unpaired t-test, and two-way ANOVA with post-hoc multiple comparisons test. Differences were considered significant if P < 0.05. Prism 7 (GraphPad Software Inc., San Diego, CA, USA) was used for analysis.

## Results

### GluD1 is expressed in NG2+ OPCs

**Fig 1:**
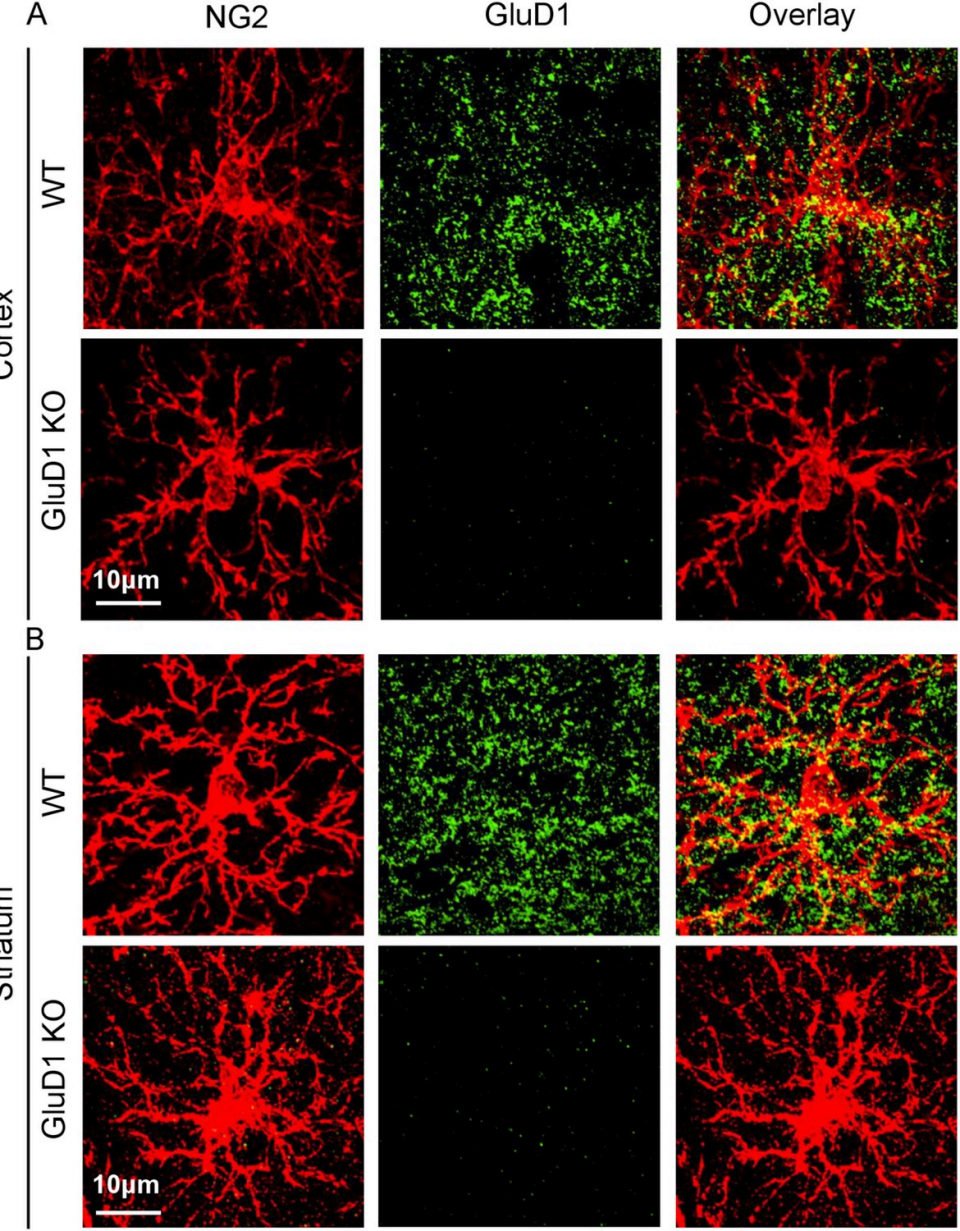
Representative images of GluD1 puncta in NG2+ cells in the cortex and striatum. Panel A shows GluD1 puncta (green) localized to NG2+ OPC (red) processes and cell body in cortex (A) and striatum (B) in wildtype. Lack of GluD1 staining in the GluD1 KO model (n = 4 mice/genotype). Scale bar = 10μm.

To characterize the expression pattern of GluD1 across glial cells including OPCs, astrocytes and microglia in different brain regions we performed colocalization studies. For this, dual immunostaining with glial cell specific markers NG2 for OPCs, GFAP for astrocytes or Iba1 for microglia and GluD1 was performed. Initially, we performed colocalization in the hippocampus and observed higher GluD1 puncta colocalized with OPC (labelled by NG2, ~29%) compared to astrocytes (labelled by GFAP, ~10%) and microglia (Iba1, ~13%) (S1 Fig in S1 File). To further corroborate these results, we performed dual immunostaining in additional regions including dorsal striatum and motor cortex as these regions are reported to have abundant OPCs and GluD1 expression [34, 49]. Similar to hippocampus, we observed higher GluD1 colocalization with NG2 compared to GFAP and Iba1 (Fig 1). Lack of staining in GluD1 KO demonstrated antibody specificity. Hence, it is evident that GluD1 is abundantly expressed in OPCs when compared to other glial cells.

### Ablation of GluD1 increases the number of OLs in the corpus callosum and motor cortex but does not alter NG2+ cells

**Fig 2:**
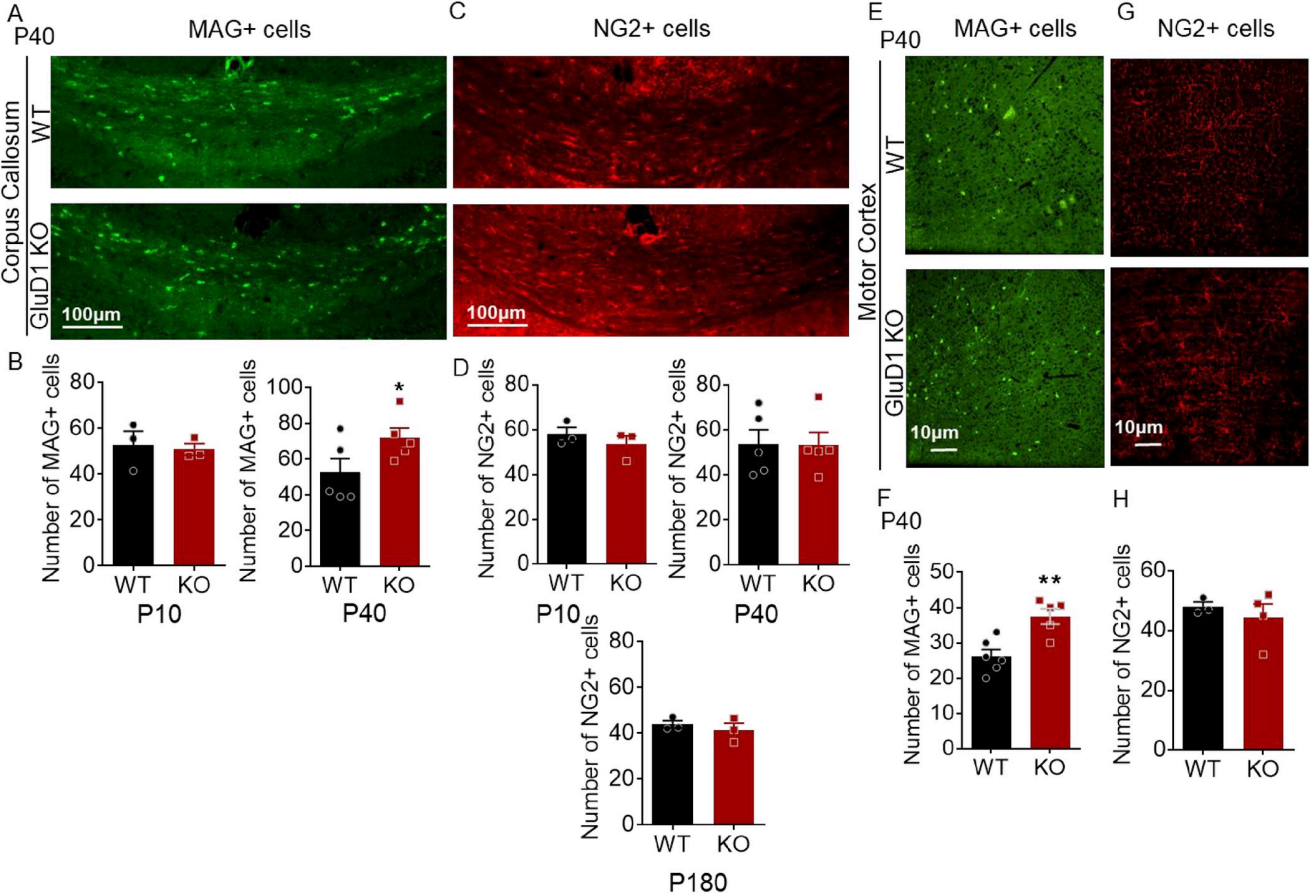
Role of GluD1 in regulating OPC differentiation. Representative images for MAG+ (A,E) and NG2+ (C,G) cells from corpus callosum (A-D) and motor cortex (E-H) of wildtype and GluD1 KO mice at P40 and its quantification. Higher number of MAG+ cells was observed in the corpus callosum (*p = 0.0405) and motor cortex (**p = 0.0037) at P40. Data were analyzed by unpaired t-test. No change in NG2+ cells in corpus callosum and motor cortex (p > 0.05). Each bar represents the mean ± SEM (n = 3–5 mice/group).

To investigate the role of GluD1 in regulating OPC differentiation we studied the changes in MAG+ cells (OL marker) and NG2+ cells in corpus callosum and motor cortex at P10, P40 and P180. The rationale behind selecting these particular time points was to address the effect of GluD1 at different developmental ages when myelination process exhibits distinct patterns. This approach allows us to capture potential differences in the effects of GluD1 ablation during critical stages of myelination, as supported by the prior work [50], which identifies key milestones in myelination and development, particularly in the forebrain during the P6–P35 period wherein myelination is at its highest rate [51] and then in subsequent 2 to 3 months myelination efficacy declines [52, 53]. We found that the number of MAG+ cells was significantly higher in corpus callosum (p = 0.0405, unpaired t-test, n = 5 mice/group, Fig 2A and 2B) and motor cortex (p = 0.0037, unpaired t-test, n = 5 mice/group, Fig 2E and 2F) of GluD1 KO mice at P40 but not at P10 as compared to wildtype mice. These results suggest that loss of GluD1 facilitate differentiation of OPCs into OLs. Next, we studied changes in NG2+ cells in GluD1 KO. No significant changes in the number of NG2+ OPCs were observed in GluD1 KO mice in corpus callosum (Fig 2C and 2D) at P10, P40 and P180 or in the motor cortex (Fig 2G and 2H) at P40, suggesting no change in proliferation of OPCs.

### Deletion of GluD1 enhances myelination in corpus callosum and motor cortex

**Fig 3:**
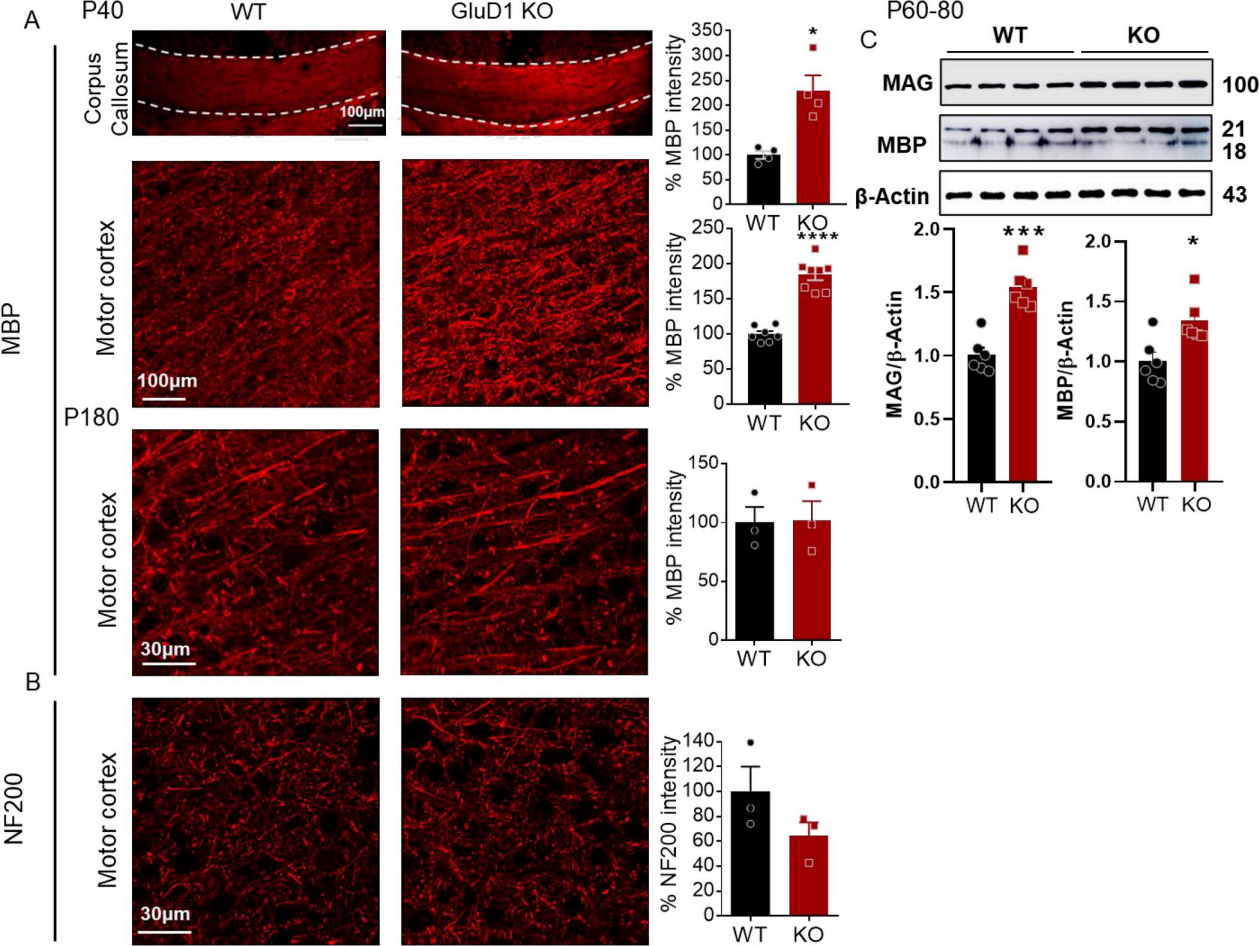
A) Role of GluD1 in regulating OPC myelination. Representative images are for myelin basic protein (MBP, A) and neurofilament (NF200, B) staining in corpus callosum and motor cortex of wildtype and GluD1 KO mice at P40 and P180 and its quantification. GluD1 KO mice showed increased MBP staining in corpus callosum (*p = 0.0196, unpaired t-test) and motor cortex (****p < 0.0001, unpaired t-test) at P40. Each bar represents the mean ± SEM (n = 4–8 mice/group). C) Immunoblot images of MAG and MBP in the somatosensory cortex of wildtype and GluD1 KO. Significant increase in MAG (***p < 0.0001, unpaired t-test) and MBP (*p < 0.0104, unpaired t-test) was found in GluD1 KO mice compared to wildtype. Data are presented as mean ± SEM (n = 6 mice/group).

To further explore whether GluD1 ablation influences OPC myelination, we performed immunohistochemical analysis for MBP (myelination marker) and NF200 (marker for myelinated axons) in the corpus callosum and motor cortex of wildtype and GluD1 KO mice. In line with the enhanced differentiation of OPCs to OLs, we observed a significant increase in MBP staining (Fig 3A) in the corpus callosum (p = 0.0196, unpaired t-test) and motor cortex (p < 0.0001, unpaired t-test) at P40 suggesting enhanced myelination in GluD1 KO mice. No significant difference was observed in MBP staining at P180 in the motor cortex. Further, examining the NF200 staining, we found a trend for lower NF200 intensity in GluD1 KO mice at P180 (Fig 3B). To confirm these results, we also performed western blot analysis for MAG and MBP in cortical synaptoneurosomes (Fig 3C). We found significant increase in MAG (p < 0.0001, unpaired t-test) and MBP (p < 0.0104, unpaired t-test) expression in GluD1 KO mice. Hence these results confirm that ablation of GluD1 in a basal setting facilitates the myelination process potentially by augmenting differentiation of OPCs to OLs.

### GluD1 ablation worsens the cuprizone-induced motor deficits

To study the effect of GluD1 ablation on cuprizone-induced motor deficits, we conducted behavioral testing following established protocol commonly used in the field. Week 0 served as a baseline measure before cuprizone treatment, while weeks 5 and 6 represent periods where the effects of demyelination and remyelination were observed. We administered a 0.2% (w/w) cuprizone diet to mice for 5 weeks, allowing the induction of acute demyelination in various CNS structures as evidenced by previous studies [54–56] followed by recovery on a control diet for a week spontaneous remyelination [57, 58]. Moreover, the rapid myelin protein re-expression within one week after withdrawal of cuprizone, as observed in the previous study [59], supports our choice to assess the effects of remyelination within a short timeframe.

**Fig 4:**
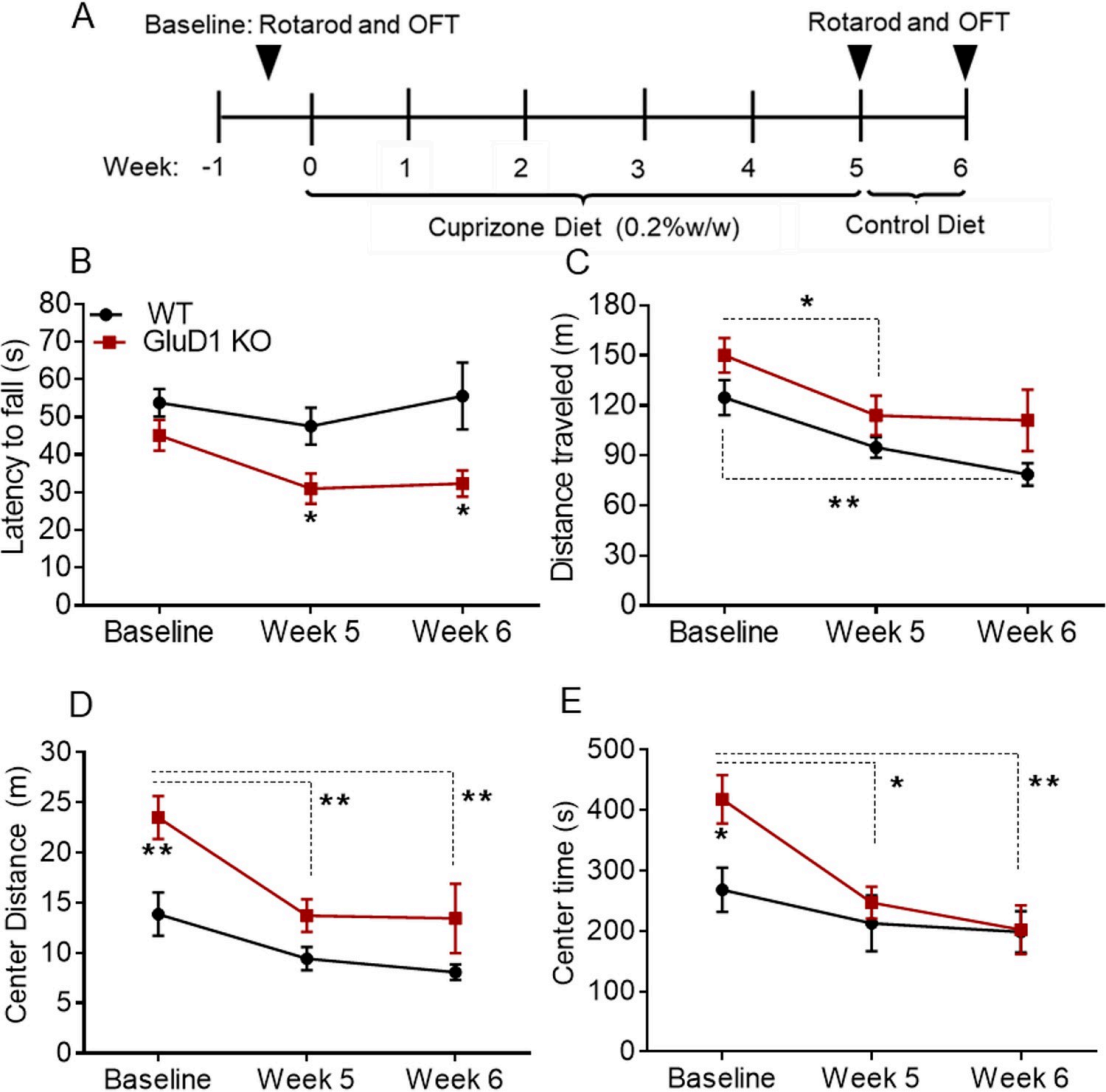
Effect of cuprizone (0.2%) diet on motor behavior in wildtype and GluD1 KO mice. A) Experimental study design indicates 0.2% cuprizone administration schedule and behavioral tests. B) In rotarod test, GluD1 KO mice showed significant reduction in the falling latency at week 5 (*p = 0.0440) and week 6 (*p = 0.0252). No significant changes in the falling latency in wildtype mice at week 5 and week 6 (both p > 0.05). Two-way ANOVA showed significant effect of cuprizone treatment on genotype [F (1, 54) = 14.83, p = 0.0003]. C) Effect of cuprizone treatment in wildtype and GluD1 KO mice on distance traveled in OFT. In the wildtype mice, a trend of reduced total distance traveled at week 5 which was aggravated at week 6 (**p = 0.0043) compared to baseline. In the GluD1 KO mice, significant reduction in total distance traveled at week 5 (*p = 0.0382) as compared to the baseline. Two-way ANOVA showed significant effect of cuprizone treatment on genotype [F (1, 50) = 8.443, p = 0.0054] and [F (2, 50) = 8.869, p = 0.0005]. D) Effect of cuprizone treatment on wildtype and GluD1 KO mice on center distance in OFT. GluD1 KO mice exhibited higher center distance during baseline (**p = 0.0012). No significant changes in center distance traveled in the wildtype mice at week 5 and week 6 (both p > 0.05). In the GluD1 KO mice, significant reduction in center distance at week 5 (**p = 0.0014) and week 6 (**p = 0.0060). Two-way ANOVA showed significant effect of cuprizone treatment on genotype [F (1, 44) = 16.27, p = 0.0002] and time [F (2, 44) = 10.82, p = 0.0002]. E) Effect of cuprizone treatment on wildtype and GluD1 KO mice on center time in OFT. GluD1 KO mice spent significant higher time in the center of OFT during baseline (*p = 0.0201). No significant changes in center time in the wildtype mice at week 5 and week 6 (both p > 0.05). In the GluD1 KO mice, significant reduction in center time at week 5 (*p = 0.0103) and week 6 (**p = 0.0057) compared to baseline. Two-way ANOVA showed significant effect of cuprizone treatment on time [F (2, 47) = 7.098, p = 0.0020]. Data represents mean ± SEM (n = 6–12 mice/group).

Motor coordination and locomotion was tested in rotarod test and open field test at week 5 (demyelination induced changes) and week 6 (remyelination induced changes, if any). In the rotarod test, cuprizone fed GluD1 KO mice had shorter latency to fall as compared to wildtype at 5 week, indicating motor coordination deficit following cuprizone treatment (p = 0.0440, Fig 4B). This effect was persistent even after feeding of control diet for one week (at week 6 time point, p = 0.0252, Fig 4B). In the open field test, we observed reduced total distance traveled at week 5 which was aggravated at week 6 compared to baseline in wildtype mice (p = 0.0043, Fig 4C). No significant changes were observed in center distance traveled, and time spent in center at week 5 and 6 compared to baseline in wildtype mice (p > 0.05, Fig 4D and 4E). In GluD1 KO mice, a reduction in total distance traveled was observed at week 5 (p = 0.0382) and remained unchanged till week 6 (Fig 4C). Additionally, significant reduction was observed in center distance (p = 0.0014, Fig 4D) and center time (p = 0.0103, Fig 3E) at week 5 compared to baseline, which persisted even after feeding of control diet for one week (at week 6; central distance p = 0.0060, central time p = 0.0057, Fig 4D and 4E). These results suggest that the ablation of GluD1 enhances susceptibility to behavioral deficits following cuprizone treatment.

### GluD1 ablation worsens the cuprizone-induced demyelination

**Fig 5:**
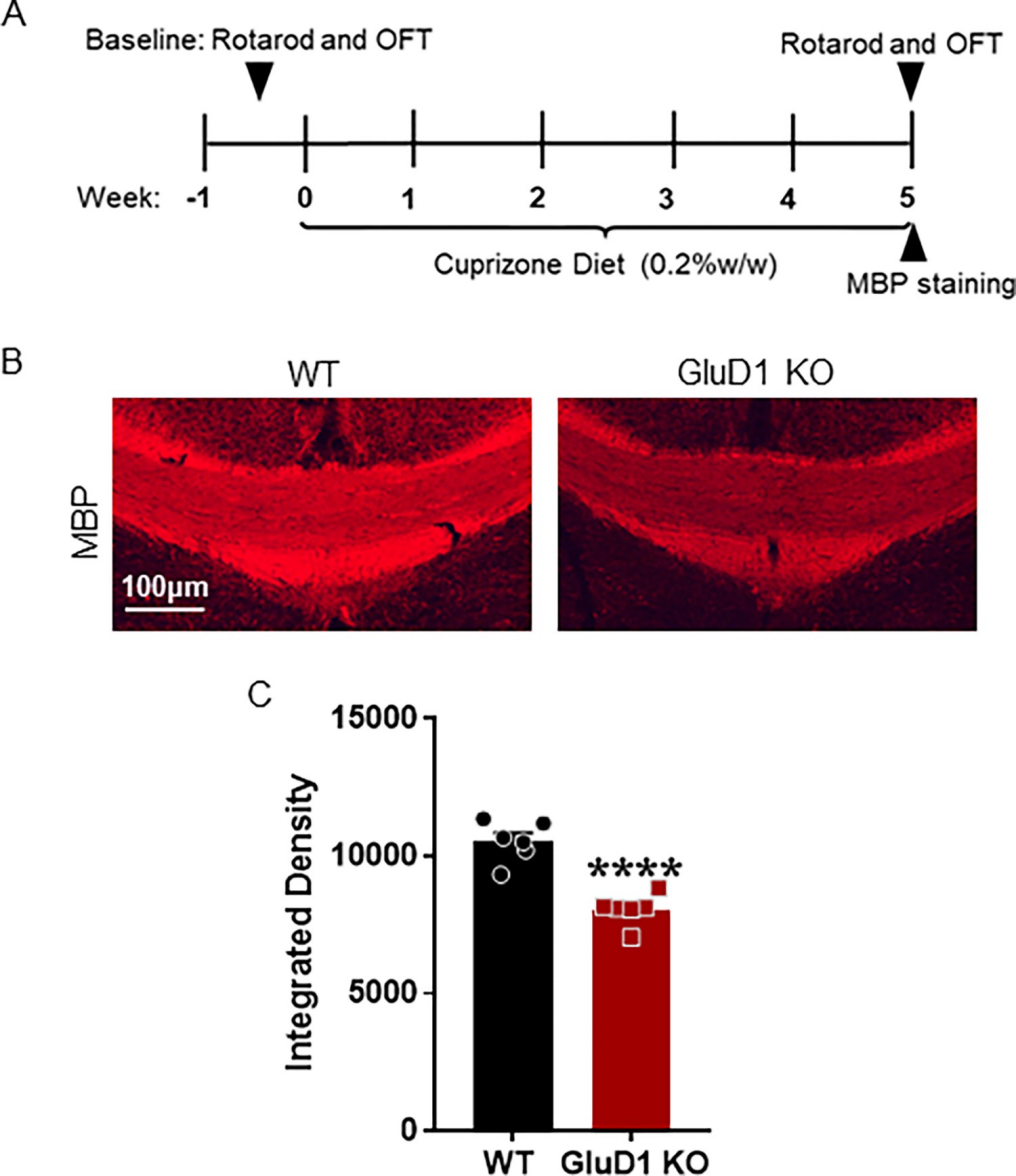
Effect of cuprizone treatment on MBP staining in corpus callosum in wildtype and GluD1 KO mice. A) Experimental study design indicates 0.2% cuprizone administration schedule and MBP staining at week 5. B) Representative images passing through corpus callosum showing MBP immunoreactivity in wildtype and GluD1 KO mice. A significant reduction in MBP staining was observed in GluD1 KO mice compared to wildtype following cuprizone treatment (****p < 0.0001, unpaired t-test). Each bar represents the mean ± SEM (n = 6 mice/group).

To understand the mechanism underlying the motor deficits in GluD1 KO mice following cuprizone treatment, we performed MBP staining to check the effects of cuprizone on myelination process. We first assessed the myelination in wildtype mice receiving control and cuprizone diet. We found decreased myelination in wildtype mice receiving cuprizone as compared to control diet (p = 0.0404, unpaired t-test, S2 Fig in S1 File). Further, we observed a severe myelin loss in GluD1 KO mice receiving cuprizone as compared to wildtype (p < 0.0001, unpaired t-test, Fig 5B and 5C). These results suggest that the ablation of GluD1 increases susceptibility to demyelination following cuprizone.

## Discussion

Present study has established a novel role of GluD1 in OPC differentiation and the process of myelination during development and following cuprizone-induced demyelination. We found that the developmental ablation of GluD1 enhances OPC differentiation and subsequent myelination process as indicated by enhanced MAG+ cells and MBP staining respectively. On the contrary, in demyelinating conditions, the ablation of GluD1 worsens motor deficits as indicated by reduced motor coordination and locomotor activity.

### GluD1 expression in OLs

Our study provides direct evidence for the expression of GluD1 in OPCs. We observed that GluD1 puncta colocalize with the OPC marker NG2 in both the motor cortex and dorsal striatum [40]. In support of our results, GluD1 mRNA is expressed in the glia, particularly OPCs (**Error! Hyperlink reference not valid.**). Importantly, when the expression of GluD1 was compared with other glial markers, such as GFAP for astrocytes and Iba1 for microglia, a significantly higher number of GluD1 puncta colocalized with OPCs (~29%) compared to astrocytes (~10%) and microglia (~13%). This finding is further supported by RNA seq studies demonstrating GluD1 expression is highest in OPCs among all CNS glial cell types [41, 60] (http://dropviz.org/). Overall, there is converging evidence of strong GluD1 expression in OPCs which may be relevant to their function.

### GluD1 regulates OPC differentiation and myelination

Myelination process involves various steps, including the proliferation, migration, and differentiation of OPCs, ultimately leading to the formation of myelin sheaths. Interestingly, OPCs have been suggested to become functionally heterogeneous both between brain regions and with age [50]. For instance, corpus callosum OPCs differentiate more efficiently into myelinating OLs than cortical OPCs [61], and despite originating from the same developmental source, gray and white matter OPCs exhibit distinct electrophysiological properties [62]. We studied the effect of GluD1 deletion on proliferation and differentiation at different developmental stages (P10, P40 and P180) and brain regions, namely motor cortex and corpus callosum. We found that the ablation of GluD1 led to an increase in the number of MAG+ cells in the corpus callosum and motor cortex at P40 but not at P10. This corresponds to the critical period window wherein the highest rate of myelination is reported [50, 51]. This increase in MAG+ cells indicates that the loss of GluD1 may facilitate the differentiation of OPCs into OLs, supporting the idea that GluD1 plays a role in regulating OPC differentiation. Interestingly, the ablation of GluD1 did not affect the number of NG2+ OPCs in either the corpus callosum or the motor cortex suggesting that the loss of GluD1 did not alter the proliferation of OPCs. Additionally, our results demonstrate that the deletion of GluD1 enhanced myelination in the corpus callosum and motor cortex as indicated by an increase in MBP at P40 in both regions but not at P180. Immunoblotting further confirmed the increase in MBP in the cortex of GluD1 KO mice. Collectively, our results support the conclusion that GluD1 may play an essential role in the developmental regulation of myelination during the critical window period.

The mechanistic target of rapamycin (mTOR) signaling pathway is known to play a role in promoting myelination in the central nervous system (CNS). It regulates OL differentiation, myelin protein expression, and myelination [63, 64]. Specifically, OL specific loss of Raptor (mTORC1) results in a delay in OL maturation in the corpus callosum [63]. Importantly mGlu5, which regulates mTOR pathway, is expressed in OPCs [41]. In our previous study, we demonstrated that GluD1 and mGlu5 colocalize and coimmunoprecipitate in the hippocampus. We found that the loss of GluD1 impairs the Homer-mGlu5 interaction and the downstream mTOR signaling pathway [65]. Additionally, the serine-threonine kinase (AKT)-mTOR phosphorylation was found to increase under basal conditions in the GluD1 KO [65, 66]. Importantly, studies have shown that, increase in mTOR activity via overexpression of constitutively active AKT or by deletion of phosphatase and tensin homolog is associated with hypermyelination in the brain [67–69]. Therefore, it is likely that the enhanced myelination observed in GluD1 KO mice could be attributed to increased AKT-mTOR signaling in OPCs which may serve as a mechanism to regulate myelination.

### Loss of GluD1 worsens cuprizone-induced deficit in motor behavior

To investigate the role of GluD1 in demyelination, we utilized the cuprizone model. Cuprizone is a toxic compound that selectively targets mature OLs in the CNS without causing damage to neurons. It produces consistent demyelination in both white and gray matter regions of the CNS [70, 71]. Mice fed with 0.2% cuprizone over 5 weeks has been shown to impair motor coordination, and causes demyelination and gliosis in the corpus callosum [72]. In the fifth week following the beginning of the cuprizone diet, when the demyelination is complete, we evaluated mice for behavioral deficits and myelin damage of the corpus callosum. Our findings revealed that, at the demyelination phase in the rotarod test cuprizone-fed GluD1 KO mice exhibited significantly shorter latency to fall than wildtype mice, indicating motor coordination deficits. Moreover, this effect persisted even after feeding a control diet for one week, indicating that GluD1 KO mice are more susceptible to long-term motor deficits following cuprizone treatment. Further, in the open field test, wildtype mice treated with cuprizone exhibited a decrease in total distance traveled compared to baseline at week 5, which persisted at week 6. However, there were no significant changes in center distance traveled and time spent in center, indicating that cuprizone treatment did not induce anxiety-like behavior in wildtype mice. Likewise, previous studies reported that cuprizone feeding decreased locomotion [73–79]. Previous studies reported a decrease of exploratory activity and motor deficits in remyelinated animals after cuprizone withdrawal which supports the findings found here [80, 81]. In contrast, GluD1 KO mice showed a significant reduction in total distance traveled at week 5, which remained unchanged at week 6. Additionally, a significant reduction was observed in center distance and center time at week 5 compared to baseline, and these behavioral deficits persisted even after feeding a control diet for one week suggesting an increase in anxiety-like behavior. Importantly, we observed significantly reduced MBP staining in cuprizone-fed GluD1 KO mice in the corpus callosum. Overall, it appears that initial insult by cuprizone is stronger in GluD1 KO mice, and the lack of behavioral improvement after returning to a normal diet for one week could be attributed to the duration of cuprizone withdrawal, as the reversal effect is dependent on the recovery time [82, 83].

Despite observed enhancement of CNS myelination in GluD1 KO mice, these mice exhibited increased susceptibility to cuprizone-induced demyelination and motor deficits. Ultrastructural localization of GluD1 using immunoperoxidase and immunogold labeling demonstrated GluD1 expression on mitochondrial membranes in dendritic shafts [40]. Interestingly, GluD2, a member of glutamate delta family, has shown to regulate mitochondria number by regulating the mitochondrial fission/fusion processes [84]. For instance, GluD2 KO mice exhibited reduced mitochondrial number and increase in mitochondrial size probably due to reduced fission and increased mitochondrial fusion [84]. Administration of cuprizone has shown to induce mitochondrial enlargement, altered metabolic rates, and impaired oxidative phosphorylation [85–88]. This mitochondrial enlargement is believed to be a compensatory response to elevated levels of reactive oxygen species (ROS) [89]. However, if ROS levels remain high for an extended period, the enlarged mitochondria become permeable and release cytochrome c and apoptosis-inducing factor-1 into the cytosol, triggering apoptosis [90, 91]. While the exact changes in mitochondrial function caused by cuprizone are not fully understood, increased mitochondrial size in demyelinated axons has been observed in various studies regardless of the demyelination cause [92–94]. OLs require a high-energy supply to synthesize the necessary lipids and proteins [95] which uphold myelin sheaths. Enhanced myelin production by mature OLs is an incredibly energy-demanding process [96, 97] which may lead to mitochondria in a hyperactive state. OLs also contain large amounts of iron stored within ferritin but have low levels of the antioxidant glutathione, indicating their vulnerability to changes in metabolic rates and oxidative stress [98, 99]. Therefore, cuprizone toxicity may cause disturbance of OL mitochondria, leading to energy shortage, ROS accumulation, and impaired lipid and protein synthesis [88, 100–102]. Thus, cuprizone-associated and hypermyelination state induced mitochondrial stress additively may aggravate the metabolic events in the OL in GluD1 KO mice leading to motor deficits and reduced myelination.

Although our study provides evidence for the involvement of GluD1 in OPC differentiation and myelination under normal and demyelinating conditions, it has certain limitations. Firstly, the potential limitation is that we do not fully understand the relationship between GluD1-mediated regulation of myelination and behavioral phenotype in GluD1 KO mice. In addition to the studies in this manuscript demonstrating a role of GluD1 in myelination there are also other roles of GluD1 that may regulate behavior, and further investigations employing conditional models are necessary to address this aspect. Secondly, we utilized global GluD1 knockout mice, which raises the possibility that the deletion of GluD1 from other cell types may also contribute to changes in OL function. Therefore, conducting in vitro OPC culture experiments would be beneficial to determine whether the observed changes in myelin markers are specifically caused by the absence of GluD1 in OPC/OL cells. Lastly, to establish more robust conclusions regarding the myelination status, incorporating electron microscopy in our research would be advantageous. This technique can provide more detailed and precise information about the myelination, thus enhancing the overall consistency of our findings.

## Supporting information

S1 File (PDF)

S1 Raw images (PDF)
