# Supplementary material for: Glutamate delta-1 receptor regulates oligodendrocyte progenitor cell differentiation and myelination in normal and demyelinating conditions
Source: PLoS One. 2023 Nov 20;18(11):e0294583. doi: 10.1371/journal.pone.0294583 (PMC10659214; doi:10.1371/journal.pone.0294583)
Supplement: S1 File — (PDF) [file pone.0294583.s001.pdf]

Supplementary material

S1 Fig.

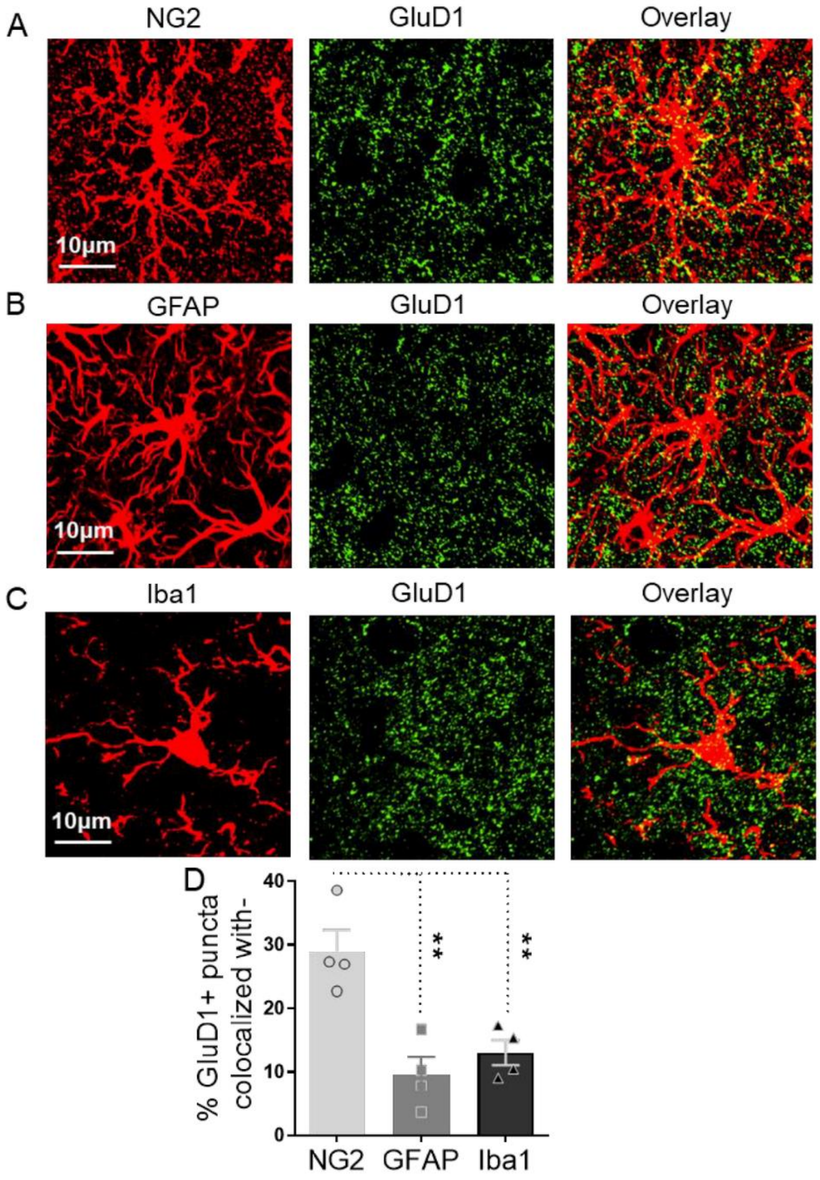

S1 Fig. Representative images of GluD1 expression in different glial cells (oligodendrocytes progenitor cells, astrocytes, and microglia) in hippocampus of wildtype mice. Immunohistochemistry was performed for GluD1 and markers for oligodendrocytes progenitor cells (OPC; NG2, A), astrocytes (GFAP, B) and microglia (Iba1, C) and its quantification in hippocampus region (D). Colocalization of GluD1 with NG2+ OPC was significantly higher as compared to Iba1 (**p = 0.0086) and GFAP (**p = 0.0024). Each bar represents the mean ± SEM (n = 4 mice/group). Scale bar = 10µm.

S2 fig.

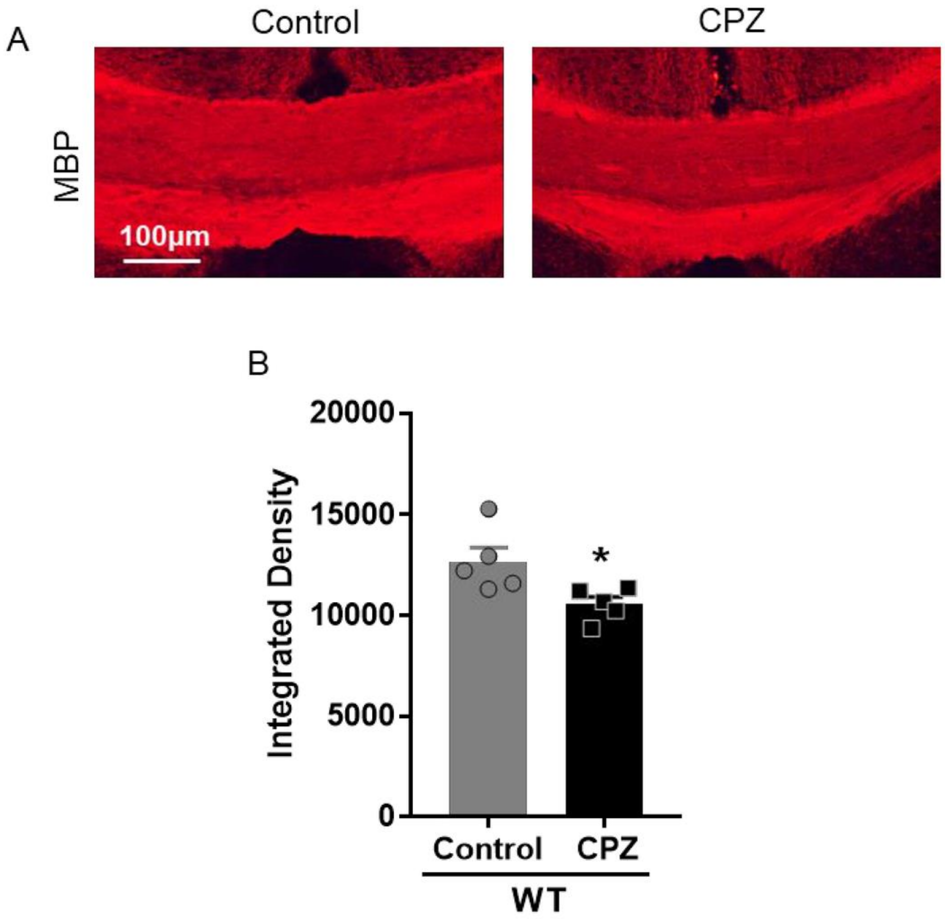

S2 Fig. Effect of cuprizone treatment on MBP staining in corpus callosum in wildtype receiving control and cuprizone diet. A) Representative images passing through corpus callosum showing MBP immunoreactivity in wildtype mice receiving control and cuprizone diet. A significant reduction in MBP staining was observed in cuprizone diet group compared to control diet group (*p = 0.0404, unpaired t-test). Each bar represents the mean ± SEM (n = 5 mice/group). Scale bar = 100 µm.
