# Supplementary figures and images for: Glutamate delta-1 receptor regulates oligodendrocyte progenitor cell differentiation and myelination in normal and demyelinating conditions
Source: PLoS One. 2023 Nov 20;18(11):e0294583. doi: 10.1371/journal.pone.0294583 (PMC10659214; doi:10.1371/journal.pone.0294583)

Figure 3C

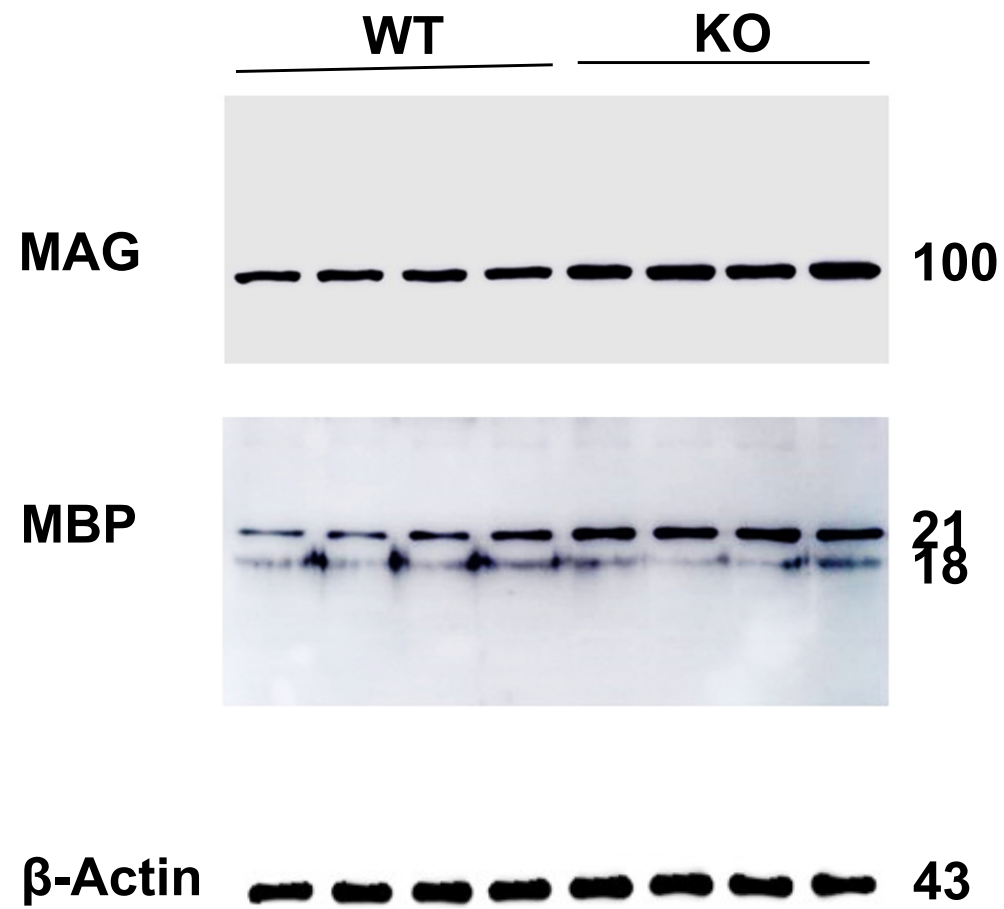

Supplement: S1 Raw images — (PDF) [file pone.0294583.s002.pdf]
